# Photochemical Implications of Changes in the Spectral Properties of Chromophoric Dissolved Organic Matter: A Model Assessment for Surface Waters

**DOI:** 10.3390/molecules28062664

**Published:** 2023-03-15

**Authors:** Nicole Altare, Davide Vione

**Affiliations:** Dipartimento di Chimica, Università di Torino, Via Pietro Giuria 5, 10125 Torino, Italy

**Keywords:** photochemical reactions, environmental photochemistry, organic matter dynamics, organic matter spectra, organic matter chromophores, photochemical fate, spectral slope

## Abstract

Chromophoric dissolved organic matter (CDOM) is the main sunlight absorber in surface waters and a very important photosensitiser towards the generation of photochemically produced reactive intermediates (PPRIs), which take part in pollutant degradation. The absorption spectrum of CDOM (*A*_CDOM_(λ), unitless) can be described by an exponential function that decays with increasing wavelength: *A*_CDOM_(λ) = 100 *d* DOC *A*_o_ e^−^ *^S^* ^λ^, where *d* [m] is water depth, DOC [mg_C_ L^−1^] is dissolved organic carbon, *A*_o_ [L mg_C_^−1^ cm^−1^] is a pre-exponential factor, and *S* [nm^−1^] is the spectral slope. Sunlight absorption by CDOM is higher when *A*_o_ and DOC are higher and *S* is lower, and vice versa. By the use of models, here we investigate the impact of changes in CDOM spectral parameters (*A*_o_ and *S*) on the steady-state concentrations of three PPRIs: the hydroxyl radical (^•^OH), the carbonate radical (CO_3_^•−^), and CDOM excited triplet states (^3^CDOM*). A first finding is that variations in both *A*_o_ and *S* have impacts comparable to DOC variations on the photochemistry of CDOM, when reasonable parameter values are considered. Therefore, natural variability of the spectral parameters or their modifications cannot be neglected. In the natural environment, spectral parameters could, for instance, change because of photobleaching (prolonged exposure of CDOM to sunlight, which decreases *A*_o_ and increases *S*) or of the complex and still poorly predictable effects of climate change. A second finding is that, while the steady-state [^3^CDOM*] would increase with increasing *A*_CDOM_ (increasing *A*_o_, decreasing *S*), the effect of spectral parameters on [^•^OH] and [CO_3_^•−^] depends on the relative roles of CDOM vs. NO_3_^−^ and NO_2_^−^ as photochemical ^•^OH sources.

## 1. Introduction

Photoinduced processes play a very important role in the degradation of biorecalcitrant contaminants in sunlit surface freshwaters [1,2,3,4]. Many contaminants of emerging concern (CECs), including several pharmaceuticals and personal care products (PPCPs), are able to survive conventional wastewater treatment due to a combination of biorecalcitrance, which slows down or prevents biodegradation by activated sludge, and water solubility, which hampers an additional pathway of elimination from the aqueous phase (partitioning on biosolids) [5,6,7,8]. These features are also an obstacle to the biological attenuation of the same CECs in natural water bodies. In these cases, photochemistry can play a key role in natural decontamination [1,2,9,10].

Photochemical reactions are usually divided into direct photolysis and indirect photochemistry. In the case of direct photolysis, pollutants absorb sunlight (i.e., radiation with λ > 280–290 nm), and the absorption process triggers transformation by ionisation, bond-breaking, or excited-state reactivity. In the case of indirect photochemistry, sunlight is absorbed by natural compounds called photosensitisers (most notably, nitrate, nitrite, and chromophoric dissolved organic matter, CDOM) that produce reactive transient species (photochemically produced reactive intermediates, or PPRIs). CECs degradation by indirect photochemistry takes place upon reaction with PPRIs, the main ones being the hydroxyl (^•^OH) and carbonate (CO_3_^•−^) radicals, the excited triplet states of CDOM (^3^CDOM*), and singlet oxygen (^1^O_2_) [11,12].

Irradiated nitrate and nitrite yield ^•^OH, which can, in turn, produce CO_3_^•−^ by oxidising HCO_3_^−^ and CO_3_^2−^. A secondary pathway to CO_3_^•−^ is the oxidation of CO_3_^2−^ by ^3^CDOM*. The irradiation of CDOM yields all the PPRIs; namely, ^3^CDOM*, ^1^O_2_, ^•^OH, and (indirectly, through ^•^OH and ^3^CDOM*) CO_3_^•−^. PPRIs are involved in pollutant degradation, but most PPRI removal from natural waters is usually accounted for by other processes not involving the contaminants. In fact, ^•^OH is mainly scavenged by DOM (dissolved organic matter, not necessarily chromophoric) and, usually to a lesser extent, by inorganic carbon (HCO_3_^−^ and CO_3_^2−^). CO_3_^•−^ is also mainly scavenged by DOM, while ^3^CDOM* in aerated waters is mostly quenched by O_2_ to produce ^1^O_2_ with ~50% yield. Finally, ^1^O_2_ is quenched by collision with the water solvent [1,13,14,15].

CDOM is the main sunlight absorber in natural waters, at least below 500 nm, which is the most important spectral range from a photochemical point of view [16]. For the same reason, CDOM is a very important photosensitiser [17,18]. The absorption spectrum of natural waters in general, and of CDOM in particular, can be described by an exponential function [19] that decreases with increasing wavelength λ [nm], as follows:

(1) $$A(\lambda )=\mathrm{DOC}\;A_{1}(\lambda )=\mathrm{DOC}\;A_{o}\;e^{-S\;\lambda }$$

here, *A*(λ) refers to an optical path length of 1 cm and has units of [cm^−1^]. Therefore, the absorbance of a water column of depth *d* [m] is given by *A_d_*(λ) = 100 *d* DOC *A*_o_ e^−*S* λ^, where 100 is the conversion factor between [m] and [cm]. Furthermore, DOC [mg_C_ L^−1^] is the dissolved organic carbon, *A*_o_ is the pre-exponential factor of the absorbance [units of L mg_C_^−1^ cm^−1^, the same as *A*_1_(λ), which is the absorbance per unit depth and DOC], and *S* [nm^−1^] is the spectral slope. As shown in Equation (1), the absorbance of CDOM at unit depth depends both on the total amount of organic matter (quantified as the DOC value) and on its spectral features (*A*_o_ and *S*). All these parameters can vary depending on environmental conditions and processes, including climate change. For instance, increased precipitation in the Scandinavian peninsula has enhanced the leaching of organic matter from soil and its transport to surface waters. At the same time, gradual recovery from acidification has progressively increased the pH of rainwater, thereby increasing the leaching efficiency of humic and fulvic acids, the ionised fractions of which are more soluble in water [20]. The resulting effect is the phenomenon of water browning [21,22,23,24] as surface waters become richer in (C)DOM. Browning has a considerable impact on the way water absorbs sunlight. Moreover, photochemical processes triggered by ^3^CDOM* and ^1^O_2_ (which are generated by irradiated CDOM) are enhanced in brownified waters at the expense of ^•^OH, CO_3_^•−^, and direct photolysis [25]. In fact, increasing DOC enhances scavenging of ^•^OH and CO_3_^•−^, while direct photolysis is inhibited as CDOM absorbs sunlight to a higher extent, which decreases the available irradiance for photolysis processes [11].

Increases in DOC from enhanced soil runoff might be observed even if average precipitation does not increase, in case intense rain events become more frequent, as predicted by climate models [26]. However, evidence suggests that increasing DOC might not always be the outcome of climate change. It has been found in several cases that inconsistent and, sometimes, negligible DOC variations have taken place alongside important changes in CDOM spectral properties, which could be quantified as modifications (either increase or decrease) of *A*_o_ and *S* [27]. To our best knowledge, quantitative photochemical implications of changes in CDOM spectral properties have not been investigated in depth, while considerable attention has been devoted to the photochemical impact of DOC changes [25,28]. We use a model approach to fill this knowledge gap and determine how changes in the spectral parameters *A*_o_ and *S* might affect the photochemistry of surface-water photosensitisers (CDOM, NO_3_^−^, and NO_2_^−^) and, therefore, steady-state concentrations of ^•^OH, CO_3_^•−^, and ^3^CDOM*. The case of ^1^O_2_ was not investigated separately because both ^1^O_2_ and ^3^CDOM* are produced by similar phenomena that involve irradiated CDOM. Furthermore, the reaction between ^3^CDOM* and O_2_ produces ^1^O_2_ with ~50% yield, while the quenching constant of ^3^CDOM* (by O_2_) is about twice higher than that of ^1^O_2_ (by collision with H_2_O). The overall outcome is [^3^CDOM*] ~ [^1^O_2_]; thus, the [^3^CDOM*] values are also representative of [^1^O_2_] [14,15].

## 2. Results and Discussion

### 2.1. Effect of CDOM Spectral Features on the Steady-State [^3^CDOM*]

The absorption spectrum of CDOM was described here by means of Equation (1). Freshwater absorbance between 290 and 500 nm is largely dominated by CDOM absorption [29]; thus, the value of *A*(λ) represents both the absorption spectrum of natural waters and that of the CDOM they contain. Representative values for surface freshwaters are *A*_o_ = 0.45 L mg_C_^−1^ cm^−1^ and *S* = 0.015 nm^−1^ [30]. However, there is environmental variability for both quantities. Moreover, climate change has the potential to alter the spectral features of CDOM, thereby modifying both *A*_o_ and *S* [27]. In order to assess the possible implications of CDOM spectral changes on the photochemistry of freshwaters, the couple (*A*_o_, *S*) = (0.45, 0.015) was taken as the central point of a 7 × 7 variation matrix, where *A*_o_ ranged from 0.30 to 0.60 L mg_C_^−1^ cm^−1^, at 0.05 steps, and *S* ranged from 0.012 to 0.018 nm^−1^, at steps of 0.001 nm^−1^. The variation intervals were chosen so as to cover a reasonable range of spectral values, valid for the majority of surface freshwaters [31].

**Figure 1:**
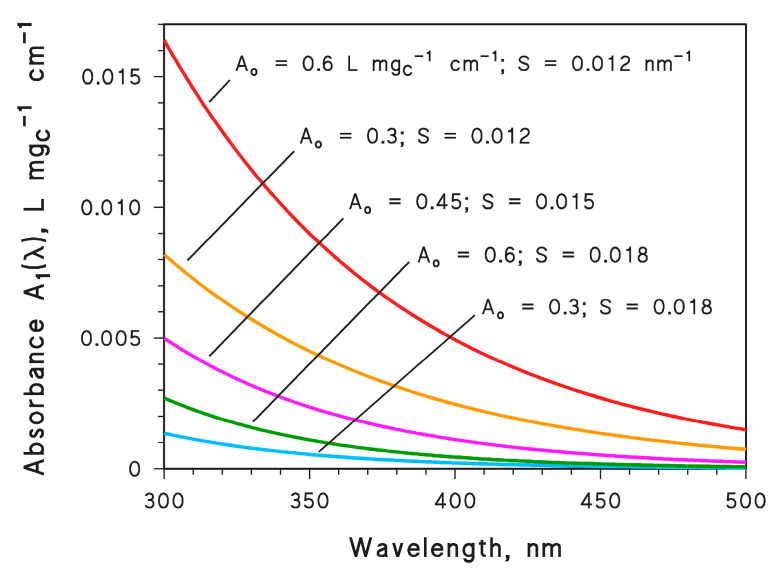
Specific water (CDOM) absorbance *A*_1_(λ) = *A*_o_ e^−^ *^S^* ^λ^ (DOC = 1 mg_C_ L^−1^, 1 cm optical path length), plotted for different values of *A*_o_ and the spectral slope *S*. Note that the measurement units are always [L mg_C_^−1^ cm^−1^] for *A*_o_ and [nm^−1^] for S.

In Figure 1, *A*_1_(λ) = *A*_o_ e^−*S* λ^ is plotted as a function of wavelength for different values of (*A*_o_[L mg_C_^−1^ cm^−1^], *S*[nm^−1^]) = (0.60, 0.012), (0.30, 0.012), (0.45, 0.015), (0.60, 0.018), and (0.30, 0.018). It is apparent that *A*_1_(λ) is higher as *A*_o_ is higher and *S* is lower. Because *S* is part of an exponent, variations in *S* have a higher impact on the values of water absorbance than variations in *A*_o_. On the one side, radiation absorption by CDOM is responsible for the photochemical generation of ^3^CDOM*, ^•^OH, and ^1^O_2_ [1,2]. On the other side, CDOM competes for sunlight irradiance with the ^•^OH sources nitrate and nitrite [11]. All these issues were taken into account by means of photochemical simulations carried out with the APEX software, which predicts the steady-state concentrations of PPRIs as a function of water chemistry, depth, and sunlight irradiance [30]. The spectral features of CDOM (both *A*_o_ and *S*) are additional input data for the software.

**Figure 2:**
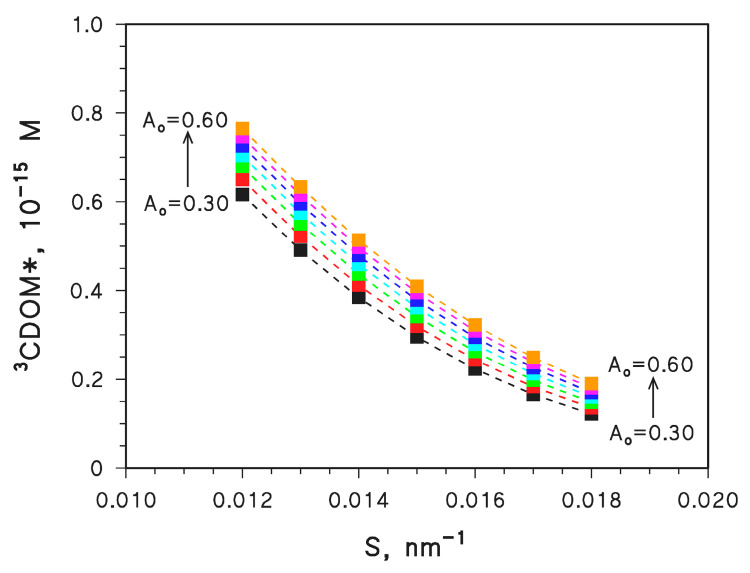
APEX-computed values of [^3^CDOM*] as a function of *S* [nm^−1^] and *A*_o_ [L mg_C_^−1^ cm^−1^]. Water conditions (reasonable values for aquatic environments [33]): 3 m depth, DOC = 5 mg_C_ L^−1^, 10^−4^ M NO_3_^−^, 10^−6^ M NO_2_^−^, 10^−3^ M HCO_3_^−^, and 10^−5^ M CO_3_^2−^. Irradiation as per fair-weather, spring equinox noon at mid latitude.

Figure 2 reports the computed steady-state [^3^CDOM*] for different values of *A*_o_ and *S*, with *d* = 3 m and DOC = 5 mg_C_ L^−1^. It is shown that [^3^CDOM*] would be higher when *A*_o_ is higher and *S* is lower, thereby following the direction of increasing CDOM absorbance (Figure 1). This is reasonable, considering that the formation rate of ^3^CDOM* from CDOM is described by the following integral equation [32]:

(2) RCDOM*3 =ΦCDOM*3 ∫λp°(λ) [1−10−ACDOM(λ)] dλ

where ΦCDOM*3  (mol Einstein^−1^) is the quantum yield of ^3^CDOM* formation, *p*°(λ) [Einstein L^−1^ s^−1^ nm^−1^] is the spectral photon flux density of sunlight, and *A*_CDOM_(λ) = 100 *d* DOC *A*_o_ e^−^ *^S^* ^λ^ (unitless). According to Equation (2), RCDOM*3  [mol L^−1^ s^−1^] increases with increasing *A*_CDOM_. Furthermore, it is clear from Figure 2 that a variation of *S* has a much larger impact on [^3^CDOM*] than a variation of *A*_o_.

The results shown in Figure 2 suggest how [^3^CDOM*] would vary at constant DOC (5 mg_C_ L^−1^) as a function of the spectral parameters *A*_o_ and *S*. The DOC value is another important factor affecting [^3^CDOM*] [11], and it is thus interesting to see how the same variation range (1 × 10^−16^ M < [^3^CDOM*] < 7.5 × 10^−16^ M; see Figure 2) could be attained by varying DOC at constant *A*_o_ and *S*. To this purpose, the DOC value was varied at fixed *A*_o_ = 0.45 L mg_C_^−1^ cm^−1^ and *S* = 0.015 nm^−1^.

**Figure 3:**
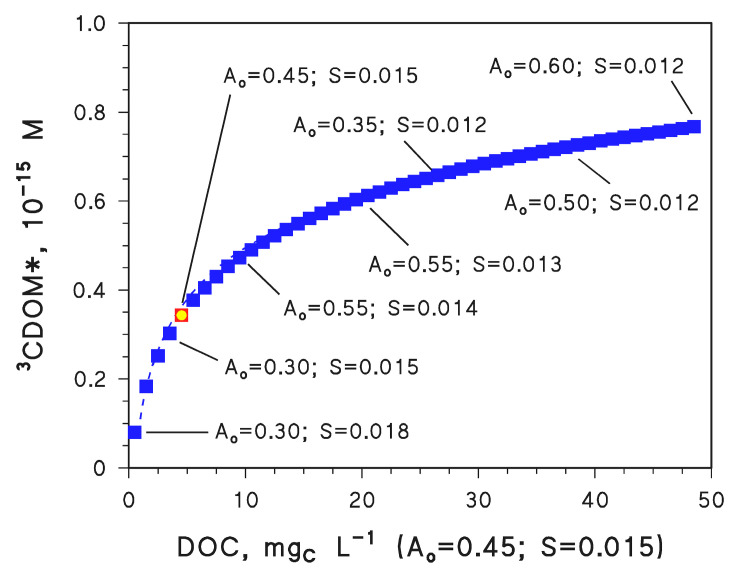
APEX-computed values of [^3^CDOM*] as a function of varying DOC, with constant *A*_o_ = 0.45 L mg_C_^−1^ cm^−1^ and *S* = 0.015 nm^−1^. The highlighted points and annotations indicate how the same values of [^3^CDOM*] were obtained with fixed DOC = 5 mg_C_ L^−1^ and different values of *A*_o_ and *S* (note that the point with *A*_o_ = 0.45 L mg_C_^−1^ cm^−1^, *S* = 0.015 nm^−1^, and DOC = 5 mg_C_ L^−1^ has a different symbol because conditions are exactly the same in both cases). Other water conditions: 3 m depth, 10^−4^ M NO_3_^−^, 10^−6^ M NO_2_^−^, 10^−3^ M HCO_3_^−^, and 10^−5^ M CO_3_^2−^. Irradiation as per fair-weather, spring equinox noon at mid-latitude.

The results of the mentioned simulations are reported in Figure 3. It is shown that to have the same [^3^CDOM*] value found for DOC = 5 mg_C_ L^−1^ and (*A*_o_, *S*) = (0.30, 0.018), one needs DOC = 0.5 mg_C_ L^−1^ if (*A*_o_, *S*) = (0.45, 0.015). At the same time, DOC = 5 mg_C_ L^−1^ and (*A*_o_, *S*) = (0.60, 0.012) give the same [^3^CDOM*] value as DOC = 50 mg_C_ L^−1^ and (*A*_o_, *S*) = (0.45, 0.015). In other words, a change Δ*A*_o_ = ±0.15 L mg_C_^−1^ cm^−1^ (i.e., ±33%) plus Δ*S* = ±0.003 nm^−1^ (i.e., ±20%) is equivalent to an order-of-magnitude variation of the DOC value. Interestingly, the DOC range 0.5–50 mg_C_ L^−1^ covers the vast majority of surface-water environments in a similar way as the studied intervals of *A*_o_ (0.30–0.60) and *S* (0.012–0.018) [31]. Therefore, CDOM spectral features have the potential to affect surface-water photochemistry in a way comparable to the DOC values.

This issue has environmental significance because, for instance, variations in either (or both) DOC values and/or CDOM spectral properties have been observed as a consequence of climate change [20,21,22,27]. It also means that both qualitative (*A*_o_, *S*) and quantitative (DOC) modifications of organic matter are potentially important for their impact on photochemical reactions. In particular, *A*_o_ depends on CDOM chromophores, while *S* is inversely related to the molecular weight of CDOM [34].

### 2.2. Effect of CDOM Spectral Features on the Steady-State [^•^OH] and [CO_3_^•−^]

The radical ^•^OH, which is also a major driver of CO_3_^•−^ production, is generated by irradiation of CDOM, nitrate, and nitrite. Because the three photosensitisers compete for sunlight irradiance, high CDOM absorbance would decrease the production of ^•^OH by both nitrate and nitrite, while favouring ^•^OH photoproduction by CDOM itself. The opposite happens when the absorbance of CDOM is low [1,2,11,12].

**Figure 4:**
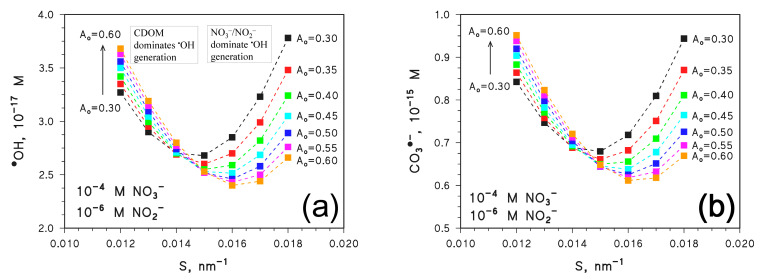
APEX-computed values of (**a**) [^•^OH] and (**b**) [CO_3_^•−^] as a function of *S* for different values of *A*_o_. Water conditions: 3 m depth, DOC = 5 mg_C_ L^−1^, 10^−4^ M NO_3_^−^, 10^−6^ M NO_2_^−^, 10^−3^ M HCO_3_^−^, and 10^−5^ M CO_3_^2−^. Irradiation as per fair-weather, spring equinox noon at mid-latitude.

Values of [^•^OH] and [CO_3_^•−^] were first calculated for the same water conditions (including constant DOC) used to derive [^3^CDOM*] in Figure 2. The results are shown in Figure 4 for different values of *A*_o_ and *S*. A trend with a minimum is observed for both [^•^OH] (Figure 4a) and [CO_3_^•−^] (Figure 4b) as a function of *S*, which is noteworthy and deserves explanation.

First of all, note that to the left-hand side of the minimum in Figure 4 (*S* < 0.015 nm^−1^, which means high *A*_CDOM_), both [^•^OH] and [CO_3_^•−^] increase with increasing *A*_o_ and decrease with increasing *S*, which is similar behaviour as that seen for [^3^CDOM*] in Figure 2. In contrast, to the right-hand side of the minimum (*S* > 0.015 nm^−1^, low *A*_CDOM_), the values of [^•^OH] and [CO_3_^•−^] decrease with increasing *A*_o_ and increase with increasing *S*.

When *A*_CDOM_ is high, ^•^OH production by nitrate and nitrite is inhibited due to light screening by CDOM. In these conditions, CDOM is the main ^•^OH source, and a further increase in *A*_CDOM_ (*A*_o_ increase, *S* decrease) mainly enhances CDOM photochemistry (inhibition of nitrate/nitrite photolysis has minor role) and leads to higher [^•^OH] and, therefore, [CO_3_^•−^]. Discussion here only focuses on ^•^OH (and CO_3_^•−^) generation because scavenging of ^•^OH and CO_3_^•−^ would not change at constant DOC, HCO_3_^−^, and CO_3_^2−^ [11,12].

When *A*_CDOM_ is low (right-hand side of the Figure 4 minimum), there is limited production of ^•^OH by CDOM and lesser inhibition of NO_3_^−^/NO_2_^−^ photolysis; thus, nitrate and nitrite play comparatively more important roles as ^•^OH sources. In these conditions, an increase in *A*_CDOM_ would mainly inhibit the photolysis of nitrate and nitrite, and the corresponding decrease in ^•^OH photogeneration by NO_3_^−^/NO_2_^−^ would not be offset by the still low (albeit enhanced) photoproduction of ^•^OH by CDOM. This issue explains why, if *S* > 0.015 nm^−1^, [^•^OH] and [CO_3_^•−^] both decrease as *A*_o_ increases and *S* decreases.

**Figure 5:**
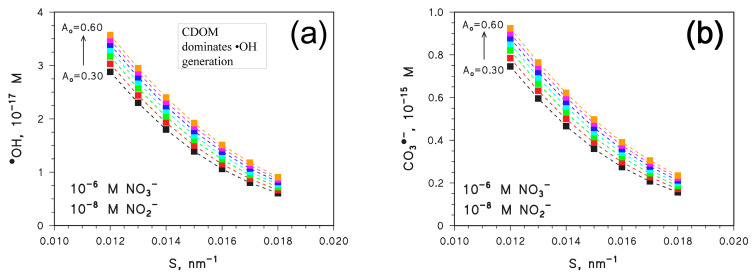
APEX-computed values of (**a**) [^•^OH] and (**b**) [CO_3_^•−^] as a function of *S* for different values of *A*_o_. Water conditions: 3 m depth, DOC = 5 mg_C_ L^−1^, 10^−6^ M NO_3_^−^, 10^−8^ M NO_2_^−^, 10^−3^ M HCO_3_^−^, and 10^−5^ M CO_3_^2−^. Irradiation as per fair-weather, spring equinox noon at mid-latitude.

To obtain better insight into the trends shown in Figure 4, the input concentration values of NO_3_^−^ and NO_2_^−^ were modified so as to make the two nitrogen species either consistently minor photosensitisers or the main sources of ^•^OH and CO_3_^•−^. Figure 5 reports the calculated steady-state [^•^OH] (Figure 5a) and [CO_3_^•−^] (Figure 5b) in the presence of 10^−6^ M NO_3_^−^ and 10^−8^ M NO_2_^−^, as a function of *S* and for different values of *A*_o_. The concentration values of NO_3_^−^ and NO_2_^−^ are 100 times lower than before, and they ensure that the photochemistry of nitrate and nitrite plays a minor role when compared to CDOM [11,12,30]. As shown in Figure 5, [^•^OH] and [CO_3_^•−^] decrease with increasing *S* and they are higher as *A*_o_ is higher. These trends resemble quite closely the trend of [^3^CDOM*] shown in Figure 2, and they mirror the photochemistry of CDOM that is enhanced as *A*_CDOM_ is higher.

The same trends also bear similarity with those reported on the left-hand side of Figure 4a,b (low *S* values). In this case as well, CDOM irradiation played the main role as the direct ^•^OH source and, indirectly, as the source of CO_3_^•−^.

**Figure 6:**
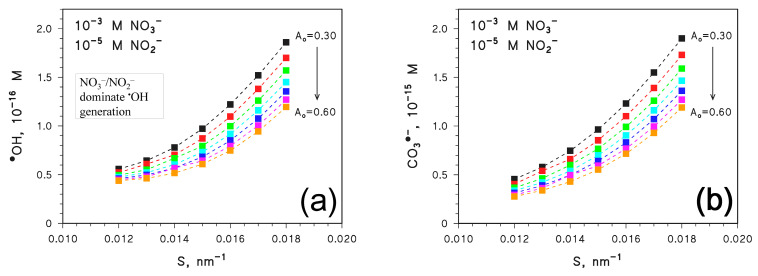
APEX-computed values of (**a**) [^•^OH] and (**b**) [CO_3_^•−^] as a function of *S* for different values of *A*_o_. Water conditions: 3 m depth, DOC = 5 mg_C_ L^−1^, 10^−3^ M NO_3_^−^, 10^−5^ M NO_2_^−^, 10^−3^ M HCO_3_^−^, and 10^−5^ M CO_3_^2−^. Irradiation as per fair-weather, spring equinox noon at mid-latitude.

The opposite case (nitrate and nitrite as major ^•^OH and CO_3_^•−^ sources) is shown in Figure 6, in which circumstance it was assumed 10^−3^ M NO_3_^−^ and 10^−5^ M NO_2_^−^. In this case, both [^•^OH] and [CO_3_^•−^] increase with increasing *S*, and they are higher as *A*_o_ is lower. Such trends resemble those of the right-hand side of Figure 4a,b (high *S* values), in that high values of [^•^OH] and [CO_3_^•−^] are obtained when *A*_CDOM_ is low. This is reasonable, because when NO_3_^−^ and NO_2_^−^ are the main sources of ^•^OH and CO_3_^•−^, the values of [^•^OH] and [CO_3_^•−^] are enhanced by a lower light-screening effect of CDOM, which produces lesser inhibition of the photochemistry of nitrate and nitrite. In these conditions, the effect of *A*_CDOM_ on CDOM photochemistry only plays a secondary role.

Low concentration values of nitrate and nitrite (Figure 5) are, for instance, representative of hypertrophic lakes, where nitrogen is the limiting element for algal growth and its inorganic forms are quickly assimilated by algae [35]. In contrast, high NO_3_^−^ and NO_2_^−^ concentrations (Figure 6) are quite near the guideline values for drinking-water quality (maximum admissible concentrations) and might apply, for instance, to a surface water body receiving inputs from contaminated groundwater [36]. Moreover, increasing *A*_o_ and decreasing *S* mean that CDOM is more aromatic and has higher molecular mass [37]. These circumstances favour sunlight absorption by CDOM (higher *A*_CDOM_ values). The opposite happens when CDOM is less aromatic and has lower molecular mass, which decreases *A*_o_ and increases *S*, causing *A*_CDOM_ to be lower. The latter circumstance may be observed when CDOM undergoes prolonged exposure to sunlight, which causes photobleaching with inactivation of chromophores (which lowers *A*_o_) and fragmentation of large molecules (which increases *S*) [34,38].

## 3. Materials and Methods

The steady-state concentrations of ^•^OH, CO_3_^•−^, and ^3^CDOM* were assessed by means of the APEX software (version 1.1 [30]), which computes PPRI concentrations as a function of solar irradiance and spectrum, water absorption spectrum, water chemistry, and depth [30,39]. The default solar irradiance in APEX is 22 W m^−2^ in the UV (290–400 nm), which can be observed in fair-weather conditions at mid-latitude during summer (15 July, 9 am or 3 pm), or at noon on the spring equinox [30]. APEX is able to compute seasonal variations in photochemical reaction rates and PPRI steady-state concentrations [40], but conditions chosen for this study were fixed and corresponded to the mid-latitude spring equinox.

Water depth *d* was fixed at 3 m, which is representative of well-illuminated water environments where photochemistry can play an important role in transformation reactions [32]. The concentration of inorganic carbon species was taken as 10^−3^ M HCO_3_^−^ and 10^−5^ M CO_3_^2−^, which is observed in several water environments [33]. In a first series of runs, it was additionally assumed DOC = 5 mg_C_ L^−1^, [NO_3_^−^] = 10^−4^ M, and [NO_2_^−^] = 10^−6^ M.

Water absorption spectrum is modelled in APEX as *A*_d_(λ) = 100 *d* DOC *A*_o_ e^−^ *^S^*^λ^, with default values of *A*_o_ = 0.45 L mg_C_^−1^ cm^−1^ and *S* = 0.015 nm^−1^ [30]. The quantum yields of ^•^OH, ^3^CDOM*, and CO_3_^•−^ photoproduction by irradiated CDOM were assumed not to vary and were left at their default values.

The APEX output data include the relative roles played by nitrate, nitrite, and CDOM in ^•^OH photoproduction [30], which largely reflect the roles of the three photosensitisers in the generation of CO_3_^•−^. The (secondary) contribution to CO_3_^•−^ from the oxidation of CO_3_^2−^ by ^3^CDOM* [41] is also taken into account by APEX.

APEX modelling assumes thoroughly mixed water environments, but it also applies to the well-mixed surface layer (epilimnion) of stratified lakes [30]. Hypolimnion photochemistry (not relevant to this study) can also be addressed by modifying the input values of *p*°(λ) [28].

## 4. Conclusions

Variations in the spectral parameters *A*_o_ and *S* strongly affect radiation absorption by CDOM. Considering the typical ranges of *A*_o_, *S*, and the DOC values that are observed in natural waters [31,33,42], it can be envisaged that the variability of CDOM spectral properties has a comparable impact as DOC variability, and cannot thus be overlooked.

The effects of spectral properties on [^3^CDOM*] depend on the resulting *A*_CDOM_ values, because [^3^CDOM*] increases as *A*_CDOM_ is higher. In contrast, variations in CDOM spectral properties affect [^•^OH] and [CO_3_^•−^] differently, depending on the relative roles of CDOM vs. NO_3_^−^/NO_2_^−^ as ^•^OH and CO_3_^•−^ sources. In particular, for a given DOC value, CDOM dominates ^•^OH and CO_3_^•−^ photoproduction when the concentration values of NO_3_^−^ and NO_2_^−^ are low, and the opposite happens when these concentrations are high [43]. At intermediates values of [NO_3_^−^] and [NO_2_^−^], the relative roles of CDOM vs. NO_3_^−^/NO_2_^−^ as ^•^OH and CO_3_^•−^ sources depend on CDOM spectral properties and, therefore, on *A*_CDOM_. High *A*_CDOM_ decreases [^•^OH] and [CO_3_^•−^] when nitrate and nitrite are the main ^•^OH/CO_3_^•−^ sources, and the opposite happens when CDOM is the main ^•^OH/CO_3_^•−^ source.

The described variations are important as far as pollutant phototransformation is concerned. In particular, ^3^CDOM* and CO_3_^•−^ are highly involved in the degradation of phenols, aromatic amines, and sulphur-containing compounds [44]. ^3^CDOM* also takes part in the transformation of phenylurea herbicides, sulphonamide antibiotics, and cyanobacterial toxins, such as microcystin-LR [44,45,46]. Highly reactive ^•^OH plays important roles in the degradation of recalcitrant pollutants such as hydrocarbons, some pesticides (e.g., atrazine), and PPCPs including carbamazepine and acesulfame K [47,48,49,50].

In environmental waters, CDOM photobleaching destroys chromophores (lower *A*_o_) and causes molecular fragmentation (higher *S*), thereby decreasing CDOM absorbance [34,37]. The steady-state [^3^CDOM*] would be decreased as a consequence, while [^•^OH] and [CO_3_^•−^] would decrease when nitrate and nitrite are low (the main effect being the inhibition of CDOM photochemistry) or increase when nitrate and nitrite are high (the main effect being enhanced photochemistry of NO_3_^−^ and NO_2_^−^, due to lower sunlight screening by CDOM). Therefore, photobleaching would amplify the photochemical role of NO_3_^−^ and NO_2_^−^ concentrations. As far as climate-change effects are concerned, variations in *A*_o_ and *S* would be highly environment-specific, and they should be assessed on a case-by-case basis.

## Data Availability

The data presented in this study are available on request from the corresponding author.

## Sample Availability

Samples of the compounds are not available from the authors.
